# Patterns, socioeconomic inequalities and determinants of healthy eating in Kenya: results from a national cross-sectional survey

**DOI:** 10.1136/bmjopen-2024-090698

**Published:** 2025-04-14

**Authors:** Elvis Omondi Achach Wambiya, Hermann P P Donfouet, Samuel Kipruto, Lyagamula Kisia, Jane Osindo, Isaac Kisiangani, James Odhiambo Oguta, Patrick G Ilboudo, Shukri F Mohamed

**Affiliations:** 1 Sheffield Centre for Health and Related Research (SCHARR), The University of Sheffield, Sheffield, UK; 2 Chronic Disease Management Unit, African Population and Health Research Center, Nairobi, Kenya; 3 World Bank, Washington, DC, USA; 4 Kenya National Bureau of Statistics, Nairobi, Kenya; 5 Emerging and Re-emerging Infectious Diseases Unit, African Population and Health Research Center, Nairobi, Kenya; 6 Maternal and Child Wellbeing Unit, African Population and Health Research Center, Nairobi, Kenya

**Keywords:** EPIDEMIOLOGY, NUTRITION & DIETETICS, PUBLIC HEALTH, Chronic Disease

## Abstract

**Abstract:**

**Objective:**

The burden of non-communicable diseases is rising in low-and-middle-income countries, with diet being a key risk factor. This study aimed to assess the patterns, socioeconomic inequalities and determinants of eating healthy in Kenya. The study is the first in Kenya to use a healthy diet index to assess dietary patterns.

**Design and methods:**

We analysed cross-sectional data from the 2015/16 Kenya Integrated Household Budget Survey. The study’s outcome variable was a continuous healthy diet index (HDI) constructed using principal component analysis from nine WHO/Food and Agriculture Organization (FAO) healthy diet recommendations. The HDI score and WHO/FAO healthy diet recommendations met were summarised for Kenyan households. Using the concentration index, we examined the socioeconomic disparities in healthy eating. In addition, multivariable linear regression was used to determine factors that influence healthy eating in Kenya.

**Results:**

A total of 21 512 households in Kenya were included, of which 60% were rural and about two-thirds headed by males. The HDI score ranged between −1.13 and 1.70, with a higher value indicating healthier eating. Overall, the average HDI score was 0.24 (95% CI: 0.24 to 0.25), interpreted as moderate. We identified key determinants including socioeconomic status and urban–rural residency differences. Healthy eating was concentrated among higher socioeconomic households, regardless of gender or location. Higher socioeconomic status (β=0.28, 95% CI 0.26 to 0.30), rural residence (β=0.18, 95% CI 0.15 to 0.20), household head being in union (β=0.04, 95% CI 0.02 to 0.06) or employed (β=0.05, 95% CI 0.02 to 0.08) were significantly associated with increased HDI scores, whereas male-headed households and lack of education were associated with significant decreases in HDI scores on average.

**Conclusions:**

Most Kenyan households do not meet all the healthy dietary recommendations, and socioeconomic inequalities exist in eating healthy. Targeted interventions that promote healthy eating based on key determinants in Kenya are required.

**STRENGTHS AND LIMITATIONS OF THIS STUDY**

- This study used a nationally representative dataset making the results generalisable to the Kenyan population.
- The generation and analysis of the healthy diet index as a continuous score reduced the potential bias of information loss in the outcome variable, which increases the validity of our results.
- Some mixed foods were excluded from the analysis because of the absence of their nutritional component values in the Kenya Food Composition Tables.
- The study being cross-sectional, we cannot infer causality on the determinants of healthy eating in Kenya.
- Despite this being the latest available survey, the fact that this survey was conducted between 2015 and 2016 means that dietary habits may have changed since and therefore may not accurately reflect the current patterns in Kenya.

## Introduction

 Non-communicable diseases (NCDs) are becoming more prevalent in low-and-middle-income countries (LMICs).^1^,^5^ NCDs have been linked to unhealthy lifestyle practices such as poor dietary habits, sedentary behaviour, cigarette use and harmful alcohol use.^6 7^ In LMICs, a nutrition transition has been documented, with shifts from traditional diets to more processed and refined foods high in saturated fats, trans fats, sugar and salt.^6^,^8^ Obesity, diabetes and cardiovascular disease are among the diet-related NCDs that have been linked to this.^9 10^ For instance, global rates of overweight and obesity have almost tripled since 1975, with LMICs accounting for about 70% of overweight or obese people.^11^ Furthermore, cardiovascular diseases and diabetes are major causes of morbidity and mortality globally and have been increasing in LMICs.^12 13^

Healthy eating habits are essential for avoiding diet-related chronic diseases. The WHO established a Global NCD Action Plan in 2013 to combat the rising global burden of NCDs.^14^ As a result, norms and criteria for eating a healthy diet have been established. The Eatwell Guide, the NOVA (not an abbreviation) classification and the WHO’s and the Food and Agriculture Organization (FAO)’s healthy diet recommendations are among them.^15^,^18^ Evidence-based interventions that promote healthy diets include salt intake reduction, replacement of trans-fat with polyunsaturated fat and public awareness on eating healthy. Despite the available standards and guidelines, increasing evidence shows poor dietary practices in LMICs.^19^ The public health implications of NCDs in these countries may become unmanageable without effective policies, initiatives and interventions.^20^

The rising burden of NCDs in developing countries has led to a growing body of research on dietary patterns and their drivers.^1 21^ Studies conducted in high-income countries (HICs) have often used a composite healthy diet index (HDI) based on WHO healthy diet guidelines to assess dietary patterns in their population.^22^,^24^ In contrast, most studies in LMICs have assessed dietary behaviour based on individual food components such as salt, sugar and fruit and vegetable consumption rather than using a composite score.^25^,^27^ Evidence from studies in LMICs has identified food costs and socioeconomic status as key determinants of dietary behaviour.^28^,^31^ Urban–rural disparities and gender differences in dietary choices have also been reported.^2532^,^34^ However, context-specific factors influencing these dietary patterns remain inadequately explored.

Despite growing research on diet and NCD prevention, few studies in Kenya have comprehensively examined healthy dietary patterns using a composite indicator. Most existing studies have focused on single dietary components, such as fruit and vegetable consumption, salt or sugar intake rather than an overall measure of healthy eating.^26 35 36^ To inform evidence-based interventions and policies aimed at promoting healthy eating, a deeper understanding of the patterns and determinants of healthy eating in Kenya is required. Socioeconomic status has been identified as a key determinant of dietary behaviour. However, socioeconomic inequalities in dietary behaviours remain underexplored. According to time preference theory, individuals with higher socioeconomic status are more likely to invest in long-term health benefits, including healthier food choices, due to lower discount rates and greater awareness of the health consequences of poor nutrition.^37^,^40^ Conversely, individuals from lower socioeconomic backgrounds may face short-term financial constraints that lead to prioritisation of cheaper, energy-dense but less nutritious foods over healthier alternatives.

To address these knowledge gaps, our study examined the patterns, socioeconomic inequalities and determinants of healthy eating in Kenya using a composite HDI. By generating this empirical evidence, this study aims to inform policy interventions and targeted programmes to improve dietary habits and reduce the burden of diet-related NCDs in Kenya.

## Methods

### Study design and participants

This study used cross-sectional data from the 2015/16 Kenya Integrated Household Budget Survey (KIHBS). The KIHBS is a nationally representative household survey that provides integrated household-level data on a wide range of indicators in order to assess the progress made in improving the living standards of the population.^41^ Data from three main tools of the KIHBS survey were used for our analysis: (1) household members’ information questionnaire, (2) household level information questionnaire and (3) household consumption expenditure information questionnaire. The household members’ questionnaire collected information on the gender of the household head, education status of the household head, age of household head and household members and marital status of the household head. The household-level questionnaire collected information on household size, residence of the household (urban/rural) and household-level expenditure on non-food items. The consumption expenditure questionnaire collected information on the types and quantities of foods consumed by Kenyan households from purchases, own production, own stock and gifts over a 7-day recall period.

### Sampling strategy

A two-stage cluster sampling design was employed to select a nationally representative random sample for the KIHBS survey. In the first stage, 2400 clusters were randomly selected comprising 988 in urban and 1412 in rural areas. In the second stage, 16 households were randomly selected per cluster, with a subsample of 10 households designated for the main KIHBS. This resulted in a final sample of 24 000 households, including 14 120 rural and 9880 urban households. Sampling weights were applied based on selection probabilities of enumeration areas, clusters and households derived from the fifth National Sample Survey and Evaluation Programme master sample.

For this study, analysis was restricted to households that reported consuming foods and dietary quantities available in the Kenya Food Composition Tables 2018 (KFCT). Households that reported consuming restaurant mixed foods, canteen foods, beer, wine, spirits, tobacco or stimulants, or narcotics, whose dietary component quantities could not be obtained, were excluded. After excluding 2488 households, the final analytical sample comprised 21 512 households (online supplemental figure 1).

### Measures

#### Outcome variable

The outcome variable for this study was a continuous HDI developed using the 2003 WHO/FAO expert recommendations on diet, nutrition and prevention of chronic diseases^18^ and the 2018 updated WHO healthy diet fact sheet.^42^ Nine dietary components and their WHO/FAO cut-off values were used to construct the composite HDI index as shown in online supplemental table 1. Nutrient composition and energy information for foods consumed in the KIHBS survey were obtained from KFCT. The KFCT lists the energy quantities (kcal) of the nutritional components per 100 g edible portion on fresh weight for commonly consumed foods in Kenya.^43^ Principal component analysis was used to generate the composite HDI from the nine dietary components.

#### Explanatory variables

Predictor variables included in the study were the household head’s gender, age, education, marital status, occupation, residence, socioeconomic status, household size and the number of members in the household in different age groups. Age was categorised into four groups: below 30 years, 30–44 years, 45–59 years and 60 years and above. The education level of the household head comprised four categories: no education, completed primary, secondary and above and other education. Marital status had two categories: in union (this included those married and cohabiting) and not in union (this included those separated/divorced, widowed and never married). Occupation of the household head was categorised into three categories: employed, self-employed and unemployed. Residence comprises rural or urban areas.

Socioeconomic status was measured using the total aggregated consumption expenditure per adult equivalent in the household. This was an aggregate measure of food and non-food consumption expenditures of the households following the best-practice guidelines provided by Deaton and Zaidi.^44^ Adult equivalents at the household level were calculated using the steps described by Smith and Subandoro.^45^ The food consumption component included expenditures of food consumed from purchases, own production, own stock and gifts over a 7-day recall period. The non-food expenditure components included household expenditure on house rent, water, electricity, gas, other cooking fuels and healthcare over the last 1 month; expenditure on clothing and footwear over the last 3 months; expenditure on education, household goods, furniture and fittings, communication, recreation and culture, insurance, financial, new/secondhand motor vehicles and accessories and miscellaneous over the last 12 months. The aggregate consumption expenditure per adult equivalent was categorised into five quintiles, that is, poorest, poor, middle, rich and richest.

### Data analysis

The sociodemographic characteristics of the study sample were described using frequencies and proportions. Counts and proportions were presented to show the distribution of categorical variables, while means and SD summarised the continuous variables. Means and 95% CI summarised the proportions of households meeting each of the healthy diet recommendations for each of the nine dietary components. The difference in proportion tests was used to assess the differences in the proportions meeting recommendations by gender of the household head, residence and socioeconomic status. Two sample t-tests were used to test differences in HDI scores by gender and residence. One-way analysis of variance (ANOVA) and Bonferroni tests were used to test differences in HDI by the different quintiles of socioeconomic status. Means, 95% CI, minimum and maximum were used to summarise the HDI index overall by gender and residence. The summary of HDI by county was mapped on the Kenyan map using ArcGIS software.

The concentration index (*CI*) was used to examine whether healthy eating is evenly distributed across poorer or richer households, with respect to gender and place of residence. To compute the *CI*, households were ranked by wealth quintiles beginning with the poorest in the population. A concentration curve (L(s)) was plotted that presents the cumulative percentage of the population ranked by wealth quintiles against their cumulative percentage of healthy and unhealthy eating.^46^,^51^ The *CI* is computed between −1 and 1, with a negative value signifying that healthy eating was concentrated among the poorest households, while a positive index value implied that healthy eating was concentrated among the richer households. When there is no inequality, the *CI* value will be zero. The *CI* is two times the area between the concentration curve (L(s)) and the diagonal, and it is given by the following formula:


(1) $$CI=\frac{2}{\mu }cov\left(FC_{i},r_{i}\right)$$

where μ is the mean of real food expenditure use; $r_{i}$ is the fractional rank of the *i*^th^ individual; $FC_{i}$ is the household consumption and cov() is the covariance. Socioeconomic inequalities nationwide, by gender and residence, were presented as the *CI* value, SE and p value. The index was considered significant if the p value was <0.05.

A multivariable linear regression model was fitted to assess factors associated with healthy diet consumption in Kenya. Crude and adjusted marginal effects and 95% CI were presented for each determinant. Variables were considered significant determinants of eating healthy if p values were <0.05. For all the analyses, survey weights were used to account for survey design and clustering. Data analysis was performed using STATA statistical software V.15.0 (StataCorp, College Station, Texas, USA).

### Patient and public involvement

Patients or community members were not involved in this study, as it was an analysis of secondary data. However, the larger study within which this analysis was embedded disseminated the results of this analysis to members of the community residing in selected counties in Kenya, namely Nairobi, Uasin Gishu, Kisumu, Isiolo and Mombasa.

## Results

### Sociodemographic characteristics of the study sample

A total of 21 512 households in Kenya were included in the sample, of which 60% were from rural areas and about two-thirds were headed by males. The average household size was 4.0 (SD 2.4) members, and the average age of household heads was 43 years (SD 15.7); male 42 years (SD 14.9) and female 46 years (SD 17.0), with about two-thirds of the study sample falling between 30 and 59 years of age. The average monthly per adult equivalent consumption expenditure was US$76.5 (SD 75.6) with 57% in the rich and richest category and 25% falling in the poor and poorest categories. About three-quarters of household heads were in union (71%) while slightly more than two-fifths had attained primary level education (44%). About half of the household heads were employed, while 41% and 11% were self-employed and unemployed, respectively (table 1).

**Table 1:** Sociodemographic characteristics of the study sample

	N	%
Monthly per adult equivalent total consumption expenditure, mean (SD) in US$	76.5 (75.6)	
Age group		
Below 30 years	3890	18.1
30–44 years	8234	38.3
45–59 years	5308	24.7
60 years and above	4080	19.0
Residence		
Urban	8556	39.8
Rural	12 956	60.2
Gender of household head		
Female	7266	33.8
Male	14 246	66.2
Education of household head		
No education	4446	20.7
Primary	9540	44.4
Secondary and above	7387	34.3
Other*	139	0.7
Employment status†		
Unemployed	2262	10.5
Employed	10 431	48.5
Self-employed	8819	41.0
Marital status of household head		
Not in union	6229	29.0
In union	15 283	71.0
Total	21 512	100.0

* Other education category comprised informal education, that is, madrassa/duksi.

† Employed comprised those in salaried employment at the public and private sector, self-employed included those with private business, and the unemployed included those with no employment. Sampling weights and clustering are used to account for the sampling design of the survey.

### Patterns of healthy diet consumption in Kenya

#### Number of dietary recommendations met by Kenyan households

Online supplemental figure 2 depicts the number of healthy diet recommendations followed by Kenyan families in general and by location. In Kenya, no household met seven or more of the healthy eating guidelines. Only 3% of Kenyan households followed six of the nine healthy eating guidelines. The majority of Kenyans (84% cumulative proportion) followed four or fewer of the healthy eating recommendations, with no household following all seven. The same was observed with regard to residence, with 82% and 86% of rural and urban households meeting four or less of the healthy diet criteria, respectively.

#### Proportion of households meeting WHO/FAO recommendations for HDI components

Online supplemental table 2 displays the percentage of Kenyan households who followed the WHO/FAO recommendations for each HDI component. The required fruit and vegetable intake was met by 45% of households, with more female-headed households (50%) and urban households (52%) meeting the recommendations than their male-headed and rural counterparts, which was statistically significant. When it came to the recommended total fat consumption, the majority of households (87%) followed the guidelines, with more female-headed (88%) and rural homes (88%) following the guidelines than male-headed (86%) and urban households (86%). Only 25% of households met the recommended total carbohydrate intake, with female-headed households (30%) and rural households (29%) being more likely to do so. Total protein and dietary fibre requirements followed a similar pattern. Overall, about a third of the households met the recommended saturated fat intake, with more urban households meeting the recommendations compared with their rural counterparts. Only 5% of households met the recommended polyunsaturated fats intake level, with more urban households meeting the recommendations compared with their rural counterparts. Overall, only 3% of households met the recommended total trans fat energy requirements, with more male-headed households meeting the recommendations. For total carbohydrates and total proteins, more than 90% of the households were above the recommended daily intake, while for polyunsaturated fats, about two-thirds were above the healthy diet recommendations (onlinesupplemental tables 3  4).

#### Distribution of HDI scores by gender, residence and socioeconomic status

The average HDI scores in the study sample are summarised in table 2 by gender, residence and socioeconomic level. The mean HDI index ranges from −1.13 to 1.70, with a higher number suggesting healthier eating habits in accordance with WHO/FAO dietary guidelines. Kenya’s average HDI score was 0.24, with urban residents scoring higher (0.25) than rural residents (0.23). There was no significant difference observed in the HDI score by gender of the household head, as observed in the overlapping confidence intervals. With regard to socioeconomic status, the findings indicate an increasing trend in the HDI score with increasing socioeconomic status, meaning that households with higher socioeconomic status were eating healthier.

**Table 2:** Summary of HDI score overall, by gender, residence and socioeconomic status

	Mean	95% CI
Overall	0.241	0.235 to 0.247
Age group		
Below 30 years	0.278	0.263 to 0.292
30–44 years	0.248	0.238 to 0.257
45–59 years	0.209	0.197 to 0.221
60 years and above	0.225	0.212 to 0.239
Residence		
Urban	0.254	0.245 to 0.264
Rural	0.231	0.223 to 0.239
Gender of household head		
Female	0.247	0.236 to 0.258
Male	0.239	0.231 to 0.246
Education of household head		
No education	0.016	0.002 to 0.030
Primary	0.240	0.231 to 0.249
Secondary and above	0.322	0.312 to 0.332
Other*	−0.046	−0.122 to 0.030
Employment status†		
Unemployed	0.140	0.120 to 0.160
Employed	0.236	0.228 to 0.245
Self-employed	0.266	0.256 to 0.275
Marital status of household head		
Not in union	0.257	0.246 to 0.268
In union	0.234	0.227 to 0.242
Socioeconomic status		
Poorest	−0.021	−0.032 to −0.010
Poor	0.170	0.157 to 0.182
Middle	0.253	0.240 to 0.266
Rich	0.340	0.327 to 0.354
Richest	0.465	0.449 to 0.480

* Other education category comprised informal education, that is, madrassa/duksi.

† Employed comprised those in salaried employment at the public and private sector, self-employed included those with private business, and the unemployed included those with no employment. Sampling weights and clustering are used to account for the sampling design of the survey.

HDI, healthy diet index.

#### Distribution of HDI score by county

**Figure 1:**
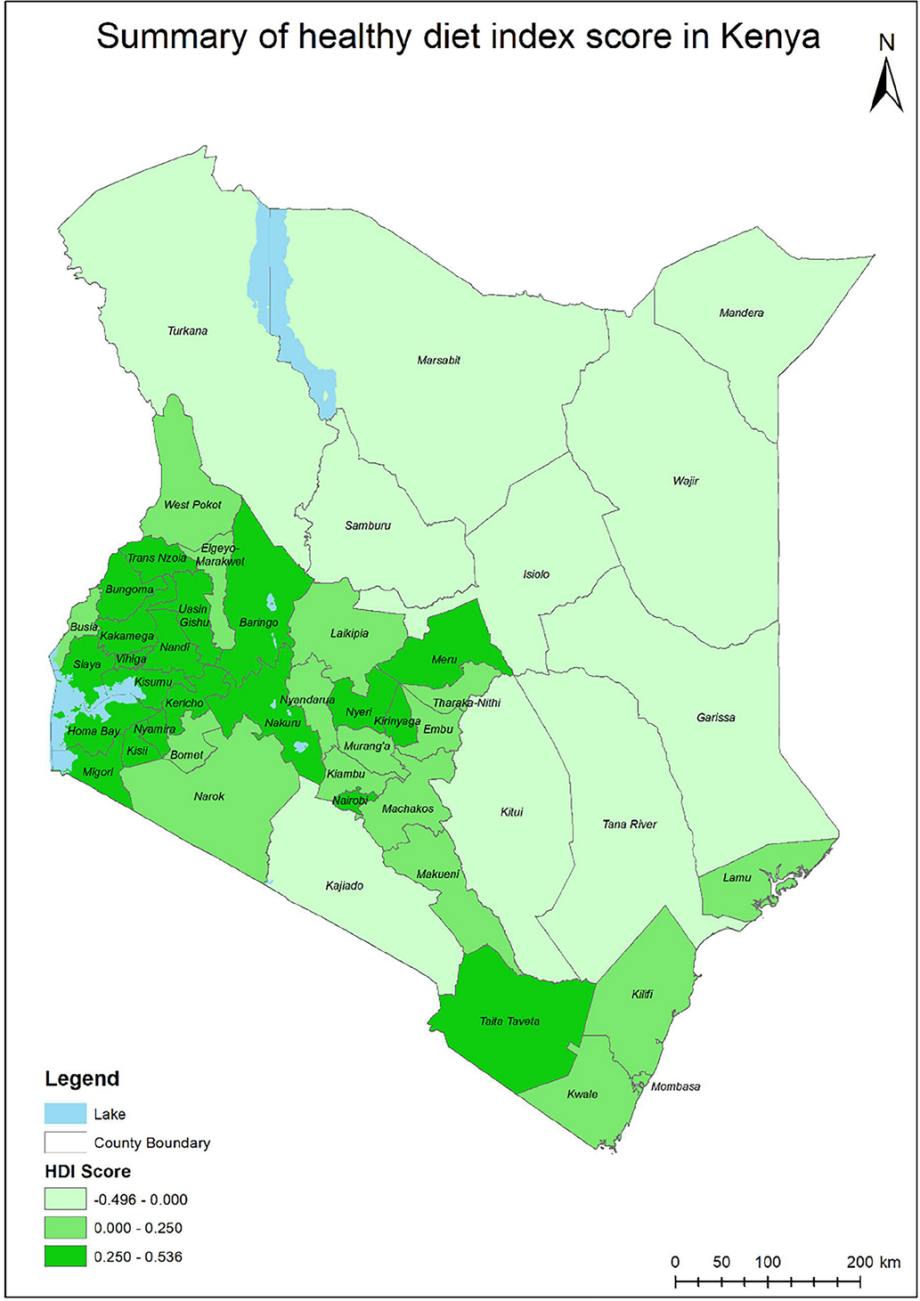
The distribution of HDI scores in Kenya by county. HDI, healthy diet index.

The distribution of the HDI scores by the 47 counties in Kenya is presented in figure 1. The results showed that Western counties had higher HDI values as compared with counties in arid and semiarid lands (ASAL) areas, which had the lowest HDI scores. It was also evident that counties that were neighbouring higher HDI counties had moderate HDI scores. Online supplemental table 5 shows the average HDI scores for each of the 47 counties.

#### Socioeconomic inequalities of eating healthy in Kenya

**Figure 2:**
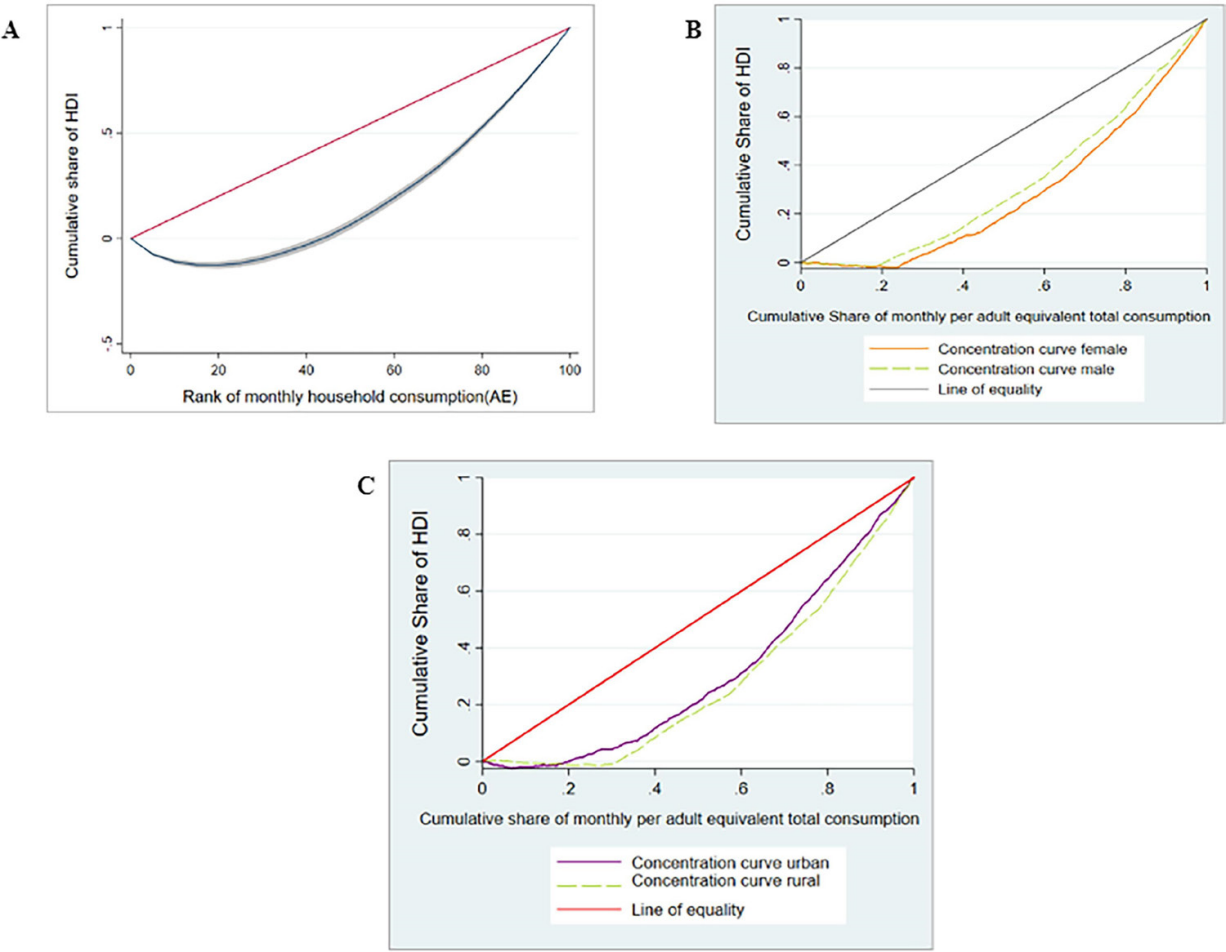
(A) Concentration curve of HDI in Kenya. (B) Concentration curve of HDI in Kenya by gender. (C) Concentration curve of HDI in Kenya by residence. HDI, healthy diet index.

Online supplemental table 6 shows the results of socioeconomic inequality analysis of HDI overall, by gender and residence, based on the *CI*. The results indicated that eating healthy foods in Kenya was concentrated among the richest households (*CI*=0.40, p<0.01). This result is confirmed by the finding in figure 2A, which shows that the concentration curve was below the line of perfect equality. Similar results (figure 2B,C) were observed with respect to gender (female *CI*=0.46, p<0.01; male *CI*=0.37, p<0.01) and residence (rural *CI*=0.50, p<0.01; urban *CI*=0.41, p<0.01). The results suggest that pro-rich inequality in eating healthy food was greater among female-headed households (diff=0.09, p<0.01) and rural households (diff=0.07, p<0.01).

### Determinants of healthy food consumption in Kenya

Table 3 presents the marginal effects from the adjusted multivariable linear regression model assessing the determinants of eating healthy in Kenya. Socioeconomic status was a strong positive predictor of healthy eating, with a one-unit increase in the log of per adult equivalent household expenditure associated with a 0.280 increase in HDI (95% CI: 0.262 to 0.297; p<0.001). The gender of the household head was significant, with male-headed households having lower HDI scores than female-headed households (β=−0.066, 95% CI: −0.087 to –0.045; p<0.001). Compared with households where the head had secondary education or higher, those with no formal education had significantly lower HDI scores (β=−0.149, 95% CI: −0.181 to –0.118; p<0.001). Similarly, households in the ‘other’ education category (including informal education) had significantly lower HDI scores (β=−0.257, 95% CI: −0.357 to –0.157; p<0.001). Compared with unemployed household heads, those who were self-employed had a moderately higher HDI (β=0.039, 95% CI: 0.009 to 0.069; p<0.01), while those employed in formal sectors had the highest HDI scores (β=0.050, 95% CI: 0.017 to 0.084; p<0.001). There were significant rural–urban differences in dietary behaviour, with rural households having higher HDI scores compared with urban households (β=0.175, 95% CI: 0.150 to 0.200; p<0.001). Finally, marital status was significantly associated with dietary behaviour. Households headed by individuals in a union had higher HDI scores compared with those headed by individuals not in a union (β=0.039, 95% CI: 0.018 to 0.061; p<0.001).

**Table 3:** Determinants of eating healthy in Kenya

	Unadjusted model		Adjusted model	
	Marginal effects	95% CI	Marginal effects	95% CI
Socioeconomic status	0.233†	0.217 to 0.250	0.280†	0.262 to 0.297
Household size	−0.031†	−0.036 to −0.027	−0.003	−0.007 to 0.000
Gender of household head				
Female (Ref)				
Male	−0.009	−0.029 to 0.012	−0.066†	−0.087 to −0.045
Log of household head age (years)	−0.057†	−0.087 to −0.028	0.091†	0.064 to 0.117
Education status of household head				
Secondary and above (Ref)				
No education	−0.262†	−0.289 to −0.235	−0.149†	−0.181 to −0.118
Primary	−0.002	−0.023 to 0.018	0.012	−0.009 to 0.032
Other	−0.288†	−0.406 to −0.170	−0.257†	−0.357 to −0.157
Occupation of household head				
Unemployed (Ref)				
Self-employed	−0.009	−0.029 to 0.012	0.039*	0.009 to 0.069
Employed	0.045†	0.024 to 0.067	0.050†	0.017 to 0.084
Residence				
Urban (Ref)				
Rural	−0.023	−0.053 to 0.007	0.175†	0.150 to 0.200
Marital status of household head				
Not in union (Ref)				
In union	−0.023*	−0.044 to −0.002	0.039†	0.018 to 0.061

Notes: Socioeconomic status = log of monthly per adult equivalent total consumption expenditure. Survey weights were used to account for the survey design and clustering.** p, *** p. Ref: Reference category. Survey weights were used to account for the survey design and clustering.

**p<0.05, ***p<0.01

* p<0.05

† p<0.01

Ref, reference category.

## Discussion

This study investigated the patterns, socioeconomic inequalities and determinants of healthy eating in Kenya. The findings reveal that only 3% of Kenyan households met six of the nine healthy eating recommendations. Furthermore, more than 80% of the population met four or fewer of the recommendations, suggesting that a significant percentage of Kenyan families are not following the full set of WHO/FAO healthy eating guidelines. Kenyan households scored moderately on the HDI, with urban households scoring higher than rural ones. We also discovered that eating healthy was associated with socioeconomic position, residence and the household head’s gender, age, occupation, marital and educational status. In the same vein, we found that healthy eating was concentrated among households with higher socioeconomic status. The findings of this study could be used to inform programmes that help Kenyan families make healthy food choices.

This study is the first in Kenya to use a healthy diet index to assess patterns of eating healthy. Previous studies conducted in HICs have used a healthy diet index.^22^,^2452^ The findings of the present study show that the majority of households were not meeting the healthy diet recommendations. This is in line with previous studies conducted in LMICs that show evidence of poor dietary behaviour.^753^,^55^ While Kenya’s vegetable and fruit intake was low, it was much higher than what was found in South Africa (32%)^56^ and what was found in an analysis that involved 52 LMICs (22%).^54^ A study in South Africa also reported that fruits were considered luxuries that were only bought if money was left over after the purchase of staple food.^57^ The low fruit and vegetable consumption observed in these countries may be linked to the low supply of fruits and vegetables that has been reported in SSA.^58^

The findings suggest substantial regional variations in eating healthy in Kenya. The Western and Central counties had the highest HDI values indicating healthier eating compared with counties in ASAL. These variations are somewhat expected because of differences in climatic conditions and social and economic factors, among other factors in different regions of Kenya.^59^

The study found that socioeconomic inequalities exist with healthy eating more concentrated among the wealthy households. This was later substantiated by the multivariate analysis, which also showed a positive and significant association between eating healthy and higher socioeconomic status. Recent global evidence indicates that healthier diets are more costly in LMICs as compared with HICs.^60 61^ Furthermore, the affordability of healthy diets in HICs including the USA and Europe is better than LMICs including parts of Asia and Africa.^30 62^ However, in both settings, higher socioeconomic status is associated with healthier dietary patterns.^29 30^ Our findings are supported by studies in other parts of Africa, including South Africa and Ghana, where socioeconomic status is an important determinant of eating healthy.^29 31^ A study conducted in Kenya showed that the foods the urban poor could afford were not sufficient for them to meet FAO dietary recommendations.^28^

Gender differences exist in choices made regarding the types of foods consumed in a household.^32^ Our study found that households headed by a female were more likely to eat healthy. This is consistent with other studies that showed that women generally make healthier food choices by eating more fruits and fibres, avoiding foods high in fats and limiting their salt intake.^32 34^ A study by Sedibe *et al*^57^ reported that female caregivers were the main promoters of healthy eating practices.

Our study also found that households headed by an educated individual were more likely to eat healthy, which corroborates findings from studies conducted in rural and urban South Africa.^63 64^ Another study in the same setting demonstrated that low education was associated with inadequate fruit and vegetable intake.^56^ Lastly, urban–rural differences have been reported in healthy diet consumption.^54^ It also emerged from our study that rural households were more likely to eat healthy compared with their urban counterparts. A systematic review and meta-analysis of salt intake in SSA found a higher consumption of salt in urban areas compared with rural areas.^25^ A study conducted in Soweto, South Africa, also demonstrated that urbanisation has led to increased consumption of diets higher in energy and containing more salt, saturated fat and sugar.^65^

The findings of this study highlight critical policy implications to improve access to and consumption of healthy foods in Kenya. Given the observed socioeconomic inequalities in healthy eating, policies should aim to address affordability barriers, particularly for low-income households. One potential strategy is to implement subsidies for fruits and vegetables, making them more accessible to households with lower socioeconomic status. This has been shown to be effective in changing population dietary behaviour.^66^ Additionally, taxation on unhealthy, ultra-processed foods such as sugar-sweetened beverages and bad fats could be explored in Kenya as it has proven effective in shifting consumer behaviour towards healthier options in other settings.^66^,^68^

The regional disparities in healthy eating patterns further emphasise the need for localised interventions. Counties in arid and semiarid regions may benefit from targeted agricultural investments, improved market infrastructure and supply chain enhancements to increase availability from other food-producing regions like the central and western region counties. Furthermore, the study’s findings on gender and education highlight the importance of integrating nutrition education into broader public health campaigns. Community-based programmes that empower male and female household heads with knowledge on nutrition and meal planning could enhance household dietary practices. Similarly, school-based initiatives that promote healthy eating from an early age may help establish long-term behavioural changes. Addressing these multifaceted challenges requires a combination of economic, agricultural and public health policies to ensure that all Kenyans, regardless of socioeconomic status or geographic location, have equitable access and consume healthy diets.

### Study strengths and limitations

A major strength of this study is the use of a nationally representative dataset, which makes our results generalisable to the Kenyan population. Second, this study provides novel evidence on patterns, determinants and socioeconomic inequalities of healthy eating in Kenya. Third, the use of the HDI as a continuous score reduced the potential bias of information loss in the outcome variable, which increased the validity of the results. However, there are some limitations to note. Some mixed foods were excluded because their nutritional values were not found in the KFCT, which may have limited the accuracy of nutrient composition data to be used in our analysis. In addition, there may have been potential for recall bias in self-reporting of dietary behaviour that could introduce inaccuracies. Because the study was cross-sectional, we cannot infer causality on the determinants of healthy eating in Kenya. The fact that the secondary data used in this study were collected between 2015 and 2016 means that dietary habits may have changed since, and the results may not accurately reflect the current consumption patterns in Kenya. However, the 2015/2016 survey is the only currently available national survey with food consumption data. The Kenya National Bureau of Statistics has piloted survey instruments for data collection for the updated survey to be conducted in the period 2024–2025.^69^ As soon as the latest data is out, we will update our analysis to capture current evidence. Despite these limitations, this research contributes to our understanding of Kenya’s healthy eating behaviours and associated determinants that can inform health policy interventions and programmes.

## Conclusions

In conclusion, the majority of Kenyan households do not meet all the nine healthy dietary recommendations, and socioeconomic inequalities exist with eating healthy concentrated among households with higher socioeconomic status. Furthermore, eating healthy is associated with higher socioeconomic status, living in a rural area and the household head being older, having education, being in employment and in union. The findings from this study could be used to inform policies and tailored interventions that promote healthy eating and the prevention of diet-related NCDs among the Kenyan population. Future research should explore the impact of specific policy interventions, such as food subsidies, taxation on unhealthy foods and nutrition education programmes, on improving dietary patterns and reducing diet-related diseases, particularly among low-income and urban households. This will benefit from updated representative surveys on food consumption to enable longitudinal tracking of changes in dietary patterns and outcomes over time.

## Supplementary material

10.1136/bmjopen-2024-090698 online supplemental figure 1

10.1136/bmjopen-2024-090698 online supplemental table 1

10.1136/bmjopen-2024-090698 online supplemental figure 2

10.1136/bmjopen-2024-090698 online supplemental table 2

10.1136/bmjopen-2024-090698 online supplemental table 3

10.1136/bmjopen-2024-090698 online supplemental table 4

10.1136/bmjopen-2024-090698 online supplemental table 5

10.1136/bmjopen-2024-090698 online supplemental table 6

## Data Availability

Data are available in a public, open access repository.
