# Supplementary figures and images for: Patterns, socioeconomic inequalities and determinants of healthy eating in Kenya: results from a national cross-sectional survey
Source: BMJ Open. 2025 Apr 14;15(4):e090698. doi: 10.1136/bmjopen-2024-090698 (PMC11997820; doi:10.1136/bmjopen-2024-090698)

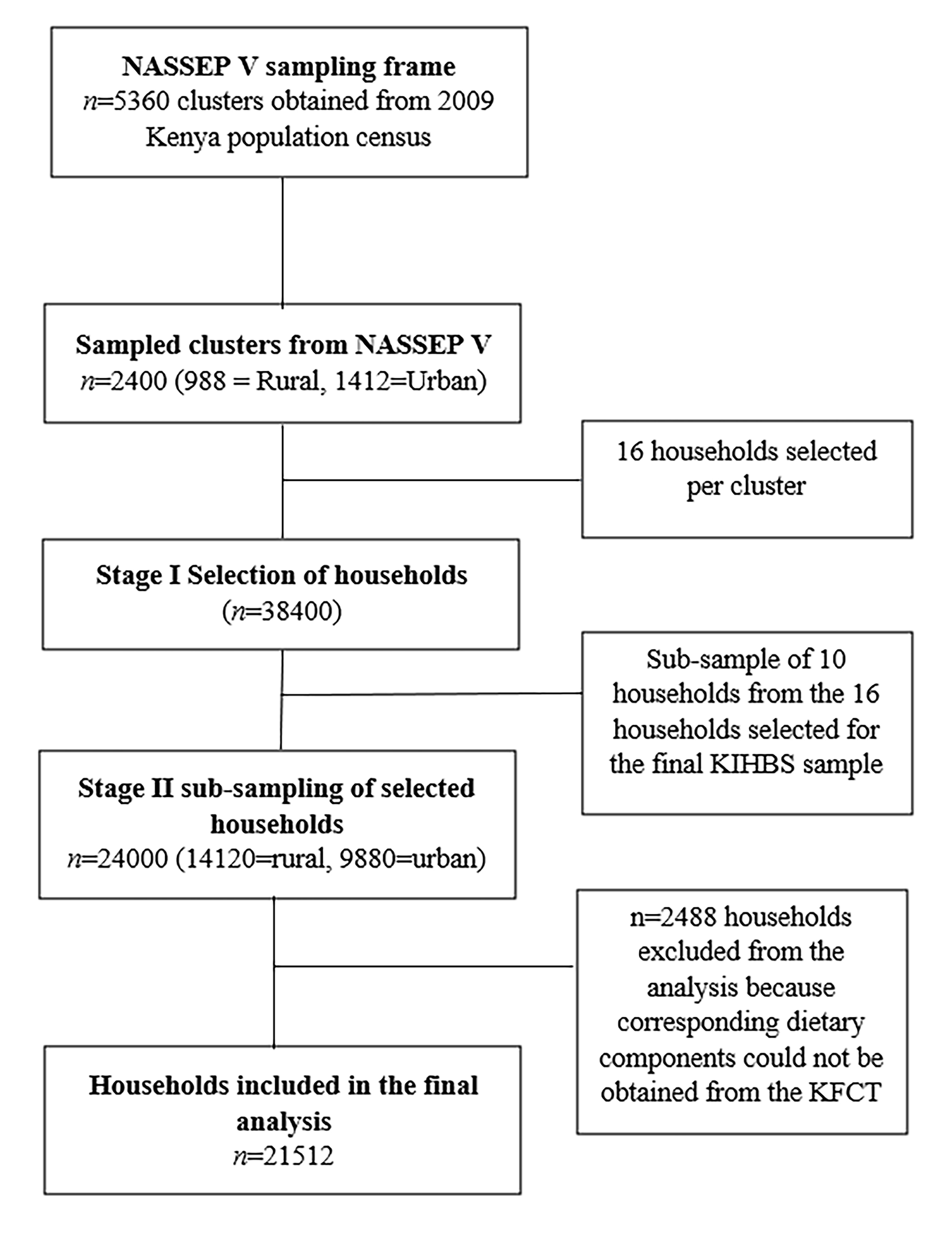

Supplement: online supplemental figure 1 [file bmjopen-15-4-s001.jpg]

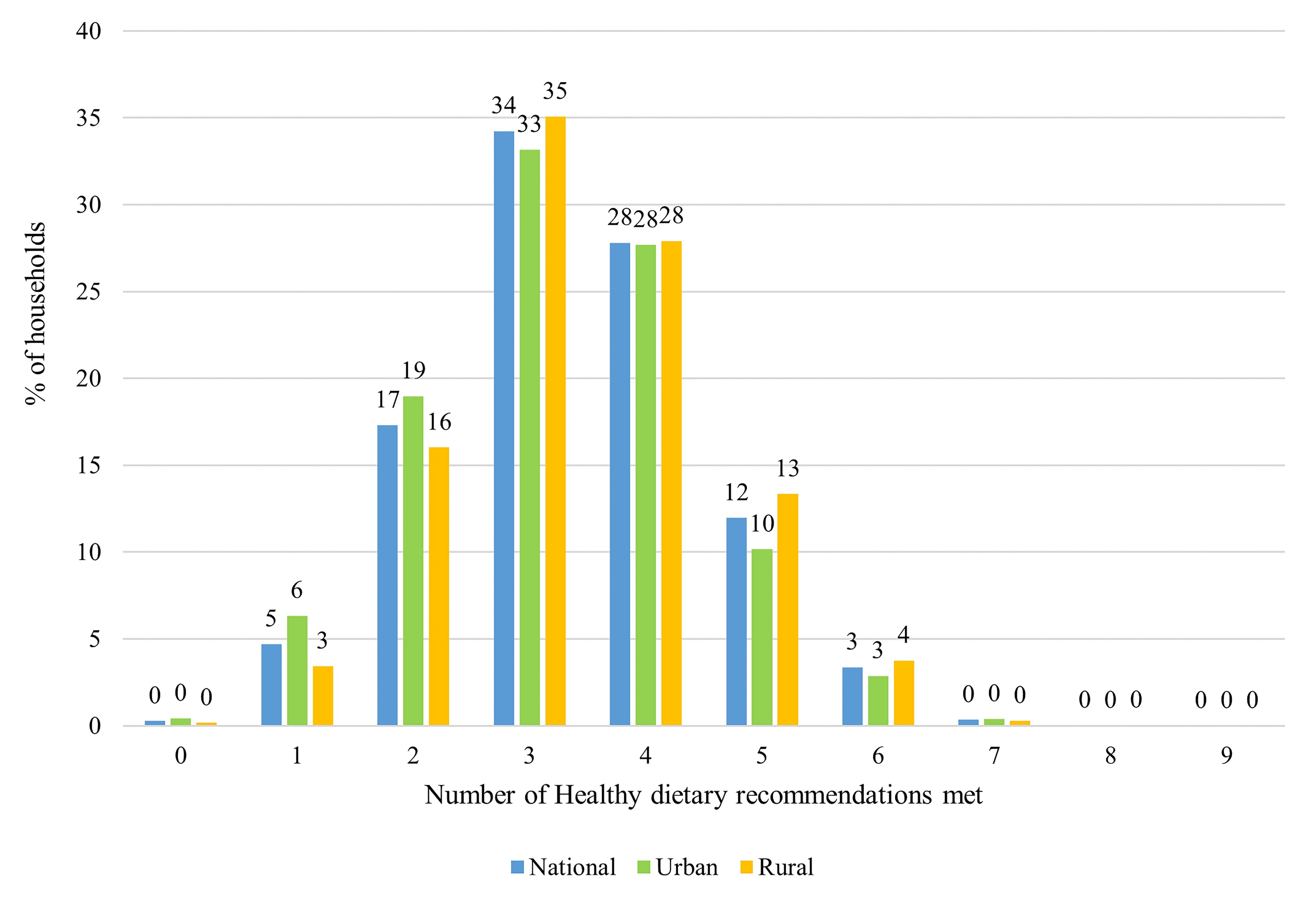

Supplement: online supplemental figure 2 [file bmjopen-15-4-s003.jpg]
