# Supplementary material for: Patterns, socioeconomic inequalities and determinants of healthy eating in Kenya: results from a national cross-sectional survey
Source: BMJ Open. 2025 Apr 14;15(4):e090698. doi: 10.1136/bmjopen-2024-090698 (PMC11997820; doi:10.1136/bmjopen-2024-090698)
Supplement: online supplemental table 1 [file bmjopen-15-4-s002.docx]

**Supplementary table 1: WHO/ FAO healthy diet recommendations for HDI dietary components**

| **Dietary factor** | **Recommendations** |
| --- | --- |
| Total fat | 15–30% |
| Saturated fatty acids (SFAs) | <10% |
| Polyunsaturated fatty acids (PUFAs) | 6–10% |
| Trans fatty acids | 1–2% |
| Total carbohydrates | 55–75% |
| Free sugars | <10% |
| Protein | 10–15% |
| Fruits and vegetables | ≥400 g/day |
| Total dietary fibre | ≥25 g/day |
| Salt intake | <5 g/day |
