# Supplementary material for: Patterns, socioeconomic inequalities and determinants of healthy eating in Kenya: results from a national cross-sectional survey
Source: BMJ Open. 2025 Apr 14;15(4):e090698. doi: 10.1136/bmjopen-2024-090698 (PMC11997820; doi:10.1136/bmjopen-2024-090698)
Supplement: online supplemental table 2 [file bmjopen-15-4-s004.docx]

**Supplementary table 2: Proportion of households meeting WHO recommendations for the dietary components of HDI**

|  |  | **Gender** | | **Residence** | | **Socioeconomic Status** | | | | |
| --- | --- | --- | --- | --- | --- | --- | --- | --- | --- | --- |
| **HDI Components** | **Overall** | **Female** | **Male** | **Urban** | **Rural** | **Poorest** | **Poor** | **Middle** | **Rich** | **Richest** |
| Fruits and vegetables, >400g per day | 45.3  (44.6 – 45.9) | 49.9  (48.7 – 51.0) | 43.1  (42.2 – 43.9) | 52.0  (51.0 – 53.1) | 40.0  (39.2 – 40.9) | 15.0  (14.2 – 15.8) | 37.3  (36.0 – 38.6) | 51.9  (50.4 – 53.5) | 63.3  (61.6 – 65.0) | 79.7  (78.0 – 81.4) |
| Total fat, 15-30% of total energy | 86.7  (86.3 – 87.2) | 88.2  (87.5 – 89.0) | 86.0  (85.5 – 86.6) | 85.6  (84.9 – 86.4) | 87.6  (87.0 – 88.2) | 85.0  (84.2 – 85.9) | 89.2  (88.3 – 90.0) | 87.9 (86.9 – 88.9) | 86.9  (85.7 – 88.1) | 84.6  (83.1 – 86.1) |
| Total carbohydrates, 55%-75% of total energy | 25.3  (24.7 – 25.9) | 30.1  (29.0 – 31.1) | 23.0  (22.3 – 23.7) | 20.8  (20.0 – 21.7) | 28.8  (28.0 – 29.5) | 30.9  (29.8 – 31.9) | 32.0  30.7 – 33.3) | 28.0  (26.6 – 29.3) | 18.7  (17.4 – 20.1) | 10.7  (9.4 – 12.0) |
| Total Protein, 10%-15% of total energy | 21.0  (20.4 – 21.5) | 24.7  (23.67 – 25.6) | 19.2  (18.5 – 19.8) | 14.3  (13.5 – 15.0) | 26.1  (25.3 – 26.8) | 36.7  (35.6 – 37.8) | 22.5  (21.4 – 23.7) | 17.0  (15.9 – 18.2) | 9.8  (8.8 – 10.9) | 9.2  (8.0 – 10.4) |
| Saturated Fats, <10% of total energy | 32.9  (32.3 – 33.6) | 33.7  (32.6 – 34.7) | 32.6  (31.8 – 33.4) | 39.8  (38.7 - 40.8) | 27.7  (26.9 – 28.4) | 35.0  (33.9 – 36.1) | 28.7  (27.5 – 30.0) | 30.8  (29.3 – 32.2) | 33.2  (31.5 – 34.8) | 37.6  (35.6 – 39.6) |
| Polyunsaturated Fats, 6%-10% of total energy | 5.0  (4.7 – 5.3) | 4.4  (4.0 – 4.9) | 5.3  (4.9 – 5.7) | 6.5  (6.0 – 7.0) | 3.9  (3.5 – 4.2) | 1.7  (1.4 – 2.0) | 4.1  (3.6 – 4.7) | 4.3  (3.6 – 4.9) | 6.4  (5.5 – 7.3) | 11.5  (10.1 – 12.8) |
| Trans-Fats, <1% of total energy | 3.0  (2.7 – 3.2) | 2.3  (2.0 – 2.6) | 3.3  (3.0 – 3.5) | 2.6  (2.3 – 3.0) | 3.2  (2.9 – 3.5) | 2.4  (2.0 – 2.7) | 3.5  (3.0 – 4.0) | 3.1  (2.5 – 3.6) | 2.4  (1.9 – 3.0) | 3.6  (2.8 – 4.4) |
| Dietary fibre, <25g/day | 71.0  (70.4 – 71.6) | 76.8  (75.8 – 77.8) | 68.2  (67.5 – 69.0) | 56.5  (55.5 – 57.6) | 82.1^***^  (81.5 – 82.8) | 7.1  (7.0 – 7.2) | 7.7  (7.6 – 7.8) | 7.0  (6.9 – 7.2) | 6.8  (6.6 – 7.0) | 6.7  (6.5 – 6.9) |
| Salt intake, <5g/ day | 45.6  (45.0 – 46.3) | 38.8  (37.6 – 39.9) | 48.9  (48.1 – 49.7) | 47.2  (46.1 – 48.2) | 44.5  (43.6 – 45.3) | 56.7  (55.6 – 57.9) | 44.6  (43.2 – 45.9) | 42.4  (40.9 – 43.9) | 39.8  (38.1 – 41.5) | 38.6  (36.6 – 40.6) |

^Notes: We are reporting the proportions and 95% CI of households who met the criteria for healthy eating based on WHO recommendations. Survey weights were used to account for the survey design and clustering. The 95% CI were computed using the Delta method.^
