# Supplementary material for: Patterns, socioeconomic inequalities and determinants of healthy eating in Kenya: results from a national cross-sectional survey
Source: BMJ Open. 2025 Apr 14;15(4):e090698. doi: 10.1136/bmjopen-2024-090698 (PMC11997820; doi:10.1136/bmjopen-2024-090698)
Supplement: online supplemental table 3 [file bmjopen-15-4-s005.docx]

**Supplementary table 3: Proportion of households above upper limit of recommended healthy diet range**

|  | **Overall** | | **Gender** | | | | **Residence** | | | |
| --- | --- | --- | --- | --- | --- | --- | --- | --- | --- | --- |
|  |  |  | **Female** | | **Male** | | **Urban** | | **Rural** | |
|  | **N** | **n (%)** | **N** | **n (%)** | **N** | **n (%)** | **N** | **n (%)** | **N** | **n (%)** |
| Total fat | 3,010 | 1496 (49.7) | 954 | 411 (43.1) | 2,056 | 1077 (52.4)*** | 1,314 | 776 (59.1)*** | 1696 | 701 (41.3) |
| Total carbohydrates | 15,534 | 15211 (97.9) | 4972 | 4943 (99.4)*** | 10,562 | 10274 (97.3) | 6392 | 6187 (96.8) | 9,142 | 9039 (98.9)*** |
| Total Protein | 16,252 | 15650 (96.3) | 5,229 | 5008 (95.8) | 11,023 | 10640 (96.5)* | 7,084 | 6850 (96.7)* | 9,168 | 8796 (95.9) |
| Polyunsaturated Fats | 20,601 | 12782 (62.0) | 6,994 | 4515 (64.6)*** | 13,607 | 8278 (60.8) | 8,088 | 5974 (73.9)*** | 12,513 | 6656 (53.2) |

Notes: N=total, n=frequency, Survey weights are used to account for the survey design and clustering
