# Supplementary material for: Patterns, socioeconomic inequalities and determinants of healthy eating in Kenya: results from a national cross-sectional survey
Source: BMJ Open. 2025 Apr 14;15(4):e090698. doi: 10.1136/bmjopen-2024-090698 (PMC11997820; doi:10.1136/bmjopen-2024-090698)
Supplement: online supplemental table 4 [file bmjopen-15-4-s006.docx]

**Supplementary table 2: Proportion of households below lower limit of recommended healthy diet range**

|  | **Overall** | | **Gender** | | | | **Residence** | | | |
| --- | --- | --- | --- | --- | --- | --- | --- | --- | --- | --- |
|  |  |  | **Female** | | **Male** | | **Urban** | | **Rural** | |
|  | **N** | **n (%)** | **N** | **n (%)** | **N** | **n (%)** | **N** | **n (%)** | **N** | **n (%)** |
| Total fat | 3,010 | 1514 (50.3) | 954 | 543 (56.9)*** | 2,056 | 979 (47.6) | 1,314 | 538 (40.9) | 1696 | 995 (58.7)*** |
| Total carbohydrates | 15,534 | 323 (2.1) | 4972 | 29 (0.6) | 10,562 | 288 (2.7)*** | 6392 | 205 (3.2)*** | 9,142 | 103 (1.1) |
| Total Protein | 16,252 | 602 (3.7) | 5,229 | 221 (4.2)* | 11,023 | 383 (3.5) | 7,084 | 234 (3.3) | 9,168 | 372 (4.1)* |
| Polyunsaturated Fats | 20,601 | 7819 (38.0) | 6,994 | 2479 (35.5) | 13,607 | 5329 (39.2)*** | 8,088 | 2114 (26.1) | 12,513 | 5857 (46.8)*** |

Notes: N=total, n=frequency, Survey weights are used to account for the survey design and clustering
