# Supplementary material for: Patterns, socioeconomic inequalities and determinants of healthy eating in Kenya: results from a national cross-sectional survey
Source: BMJ Open. 2025 Apr 14;15(4):e090698. doi: 10.1136/bmjopen-2024-090698 (PMC11997820; doi:10.1136/bmjopen-2024-090698)
Supplement: online supplemental table 5 [file bmjopen-15-4-s007.docx]

**Supplementary table 5: Average HDI scores in Kenya by county**

| **County** | **HDI score** |
| --- | --- |
| Kakamega | 0.536 |
| Bungoma | 0.503 |
| Kisii | 0.451 |
| Vihiga | 0.428 |
| Homa Bay | 0.409 |
| Nakuru | 0.370 |
| Nandi | 0.369 |
| Kirinyaga | 0.366 |
| Migori | 0.359 |
| Trans Nzoia | 0.359 |
| Taita/Taveta | 0.351 |
| Nairobi | 0.335 |
| Nyeri | 0.310 |
| Nyamira | 0.310 |
| Baringo | 0.293 |
| Kericho | 0.285 |
| Siaya | 0.280 |
| Meru | 0.272 |
| Kisumu | 0.255 |
| Uasin Gishu | 0.253 |
| Narok | 0.250 |
| Tharaka-Nithi | 0.248 |
| Busia | 0.227 |
| Embu | 0.215 |
| Bomet | 0.215 |
| Lamu | 0.212 |
| Murang'a | 0.206 |
| Kilifi | 0.199 |
| Kiambu | 0.186 |
| Machakos | 0.181 |
| Nyandarua | 0.164 |
| Laikipia | 0.159 |
| Kwale | 0.155 |
| Elgeyo Marakwet | 0.139 |
| West Pokot | 0.103 |
| Makueni | 0.095 |
| Mombasa | 0.088 |
| Kajiado | -0.030 |
| Kitui | -0.050 |
| Samburu | -0.063 |
| Isiolo | -0.159 |
| Turkana | -0.167 |
| Tana River | -0.192 |
| Marsabit | -0.283 |
| Mandera | -0.333 |
| Garissa | -0.397 |
| Wajir | -0.496 |
