# Supplementary material for: Patterns, socioeconomic inequalities and determinants of healthy eating in Kenya: results from a national cross-sectional survey
Source: BMJ Open. 2025 Apr 14;15(4):e090698. doi: 10.1136/bmjopen-2024-090698 (PMC11997820; doi:10.1136/bmjopen-2024-090698)
Supplement: online supplemental table 6 [file bmjopen-15-4-s008.docx]

**Supplementary table 5: Concentration index (CI) values of HDI countrywide, by gender and residence**

|  | **CI value** | **S.E.** | **p-value** |
| --- | --- | --- | --- |
| **Overall** | 0.403 | 0.014 | 0.000 |
| **Gender** |  |  |  |
| Female | 0.464 | 0.020 | 0.000 |
| Male | 0.374 | 0.016 | 0.000 |
| **Residence** | |  |  |
| Urban | 0.414 | 0.021 | 0.000 |
| Rural | 0.489 | 0.013 | 0.000 |

Notes: Survey weights were used to account for the survey design and clustering.
