# Supplementary material for: Patterns, socioeconomic inequalities and determinants of healthy eating in Kenya: results from a national cross-sectional survey
Source: BMJ Open. 2025 Apr 14;15(4):e090698. doi: 10.1136/bmjopen-2024-090698 (PMC11997820; doi:10.1136/bmjopen-2024-090698)
Supplement: Supplementary file 9 [file bmjopen-2024-090698.reviewer_comments.pdf]

PEER REVIEW HISTORY

BMJ Open publishes all reviews undertaken for accepted manuscripts. Reviewers are asked to complete a checklist review form and are provided with free text boxes to elaborate on their assessment. These free text comments are reproduced below.

ARTICLE DETAILS

Title (Provisional)

Patterns, socioeconomic inequalities, and determinants of healthy eating in Kenya: Results from a national cross-sectional survey

Authors

Wambiya, Elvis Omondi Achach; Donfouet, Hermann P.P.; Kipruto, Samuel; Kisia, Lyagamula; Osindo, Jane; Kisiangani, Isaac; Oguta, James; Ilboudo, Patrick G.; Mohamed, Shukri F

VERSION 1 - REVIEW

**Reviewer**	**1**
**Name**	**Tafadzwa Lukwa, Akim**
**Affiliation**	**University of Cape Town, Health Economics Unit**
**Date**	**26-Oct-2024**
**COI**	**None**

Thank you for submitting your manuscript to BMJ Open and allowing me to review your work. The study "Patterns, Socioeconomic Inequalities, and Determinants of Healthy Eating in Kenya: Results from a National Cross-Sectional Survey" addresses a significant and timely public health issue with implications for nutritional policy and intervention in Kenya. The thorough approach to analyzing the determinants of healthy eating within the socio-economic framework of Kenya provides valuable insights that could potentially influence public health strategies and dietary guidelines. Below, I offer some suggestions that could further strengthen the manuscript and enhance its contribution to the literature.

Title

• Original Line: 1

• Suggestion: Specify the dietary components in the title to clarify the focus of healthy eating. For example:

o Revised Title: "Patterns, Socioeconomic Inequalities, and Determinants of Fruit and Vegetable Intake in Kenya: A National Cross-Sectional Survey Analysis"

o Rationale: This adjustment clarifies the dietary focus, enhancing relevance and searchability for readers interested in diet-related aspects.

Abstract

• Lines: 31-36

• Modifications:

o Objective: Add specifics to the dietary behaviors studied, such as "This study aims to assess the patterns and socioeconomic determinants of fruit and vegetable intake among Kenyan households."

o Methods: Include key statistical tools used, "utilizing principal component analysis and multivariable linear regression."

o Results: Briefly highlight significant findings, e.g., "We identified key determinants including socioeconomic status and urban-rural residency differences."

o Rationale: These modifications enhance the abstract's clarity and informativeness, providing a concise snapshot of the study's scope, methods, and key findings.

Introduction

• Lines: 4-5

• Enhancements:

o Elaborate on prior studies specific to the region to better frame the research within the existing literature.

o Explicitly state the research gap your study addresses, such as limited data on the impact of socioeconomic factors on diet within Kenya.

o Example: "While numerous studies have documented dietary habits in East Africa, few have dissectively analyzed the socioeconomic gradients impacting dietary quality among Kenyan households."

Methods

• Lines: 5-6

• Details to Add:

o Clarify the sampling methodology, "employing stratified random sampling to ensure diverse representation from different Kenyan counties."

o Proactively mention methodological constraints that might impact results, like "potential recall bias in dietary self-reporting and limitations in the accuracy of food composition data for local dishes."

o Rationale: Provides greater transparency about the methodology's robustness and its limitations.

Results

• Lines: 13-14

• Enhancements:

o Introduce a summary table of key quantitative findings, such as average HDI scores across demographic groups.

o Clarify significance levels in the results discussion, noting specific p-values or confidence intervals where applicable.

o Rationale: Facilitates quick data assimilation and underscores the statistical rigor of the findings.

Discussion

• Lines: 18-19

• Improvements:

o Compare your findings with similar studies globally, discussing similarities or differences and what these might mean in the Kenyan context.

o Address the implications of your findings for public health policy in Kenya, proposing specific interventions or policy adjustments.

o Rationale: Contextualizes the findings within a broader international scope and links results to practical, actionable public health strategies.

Conclusion

• Lines: 20-21

• Suggested Additions:

o Concisely reiterate main findings and their implications for future research or policy.

o Recommend directions for future research, such as longitudinal studies to track dietary pattern changes and health outcomes over time.

o Rationale: Enhances the manuscript's impact by highlighting its implications and encouraging further investigation.

**Reviewer**	**2**
**Name**	**Thomas, Roger**
**Affiliation**	**University of Calgary, Family Medicine**
**Date**	**17-Dec-2024**

COI **None**

This is an important study.

Introduction

Please provide the search terms and databases for your literature review so that readers can assess the comprehensiveness of your search.

You wrote: "In LMICs, a nutrition transition has been documented, with shifts from traditional diets to more processed and refined foods high in saturated fats, trans fats, sugar, and salt 5-7. Obesity, diabetes, and cardiovascular disease are among the diet-related NCDs that have been linked to this 8,9."

[Your readers will be be very interested in numerical outcomes. Please provide these for comparison to your study].

You wrote: "Despite the available standards and guidelines, increasing evidence shows poor dietary practices in LMICs 15"

"The rising burden of NCDs in developing countries has led to generation of more evidence on dietary patterns and their drivers 1,17. Studies conducted in high-income countries (HICs) have used a composite healthy diet indicator from WHO healthy diet guidelines to assess dietary patterns in their population 18-20."

[please provide detailed numerical outcome data so readers can understand the dimensions of the problem]

Methods

You wrote: "A two-stage cluster sampling design was employed to select a representative sample. In the first stage, 2,400 (988 in urban and 1,412 in rural areas) clusters were selected and 16 households were selected in the second stage while a sub-sample of 10 households was conducted for the main KIHBS."

[so no randomisation was used. Please provide numerical data to demonstrate whether your sample reflects accurately the Kenyan population]

Results

You wrote: "The average monthly per adult equivalent consumption expenditure was USD 76.5 (SD 75.6) with 57% in the rich and richest category and 25% falling in the poor and poorest categories. Majority of household heads had up to primary level education (65%) and were in union (71%). About half of the household heads were employed while 41% and 11% were self-employed …."

"The findings indicate that a high percentage of Kenyan families were not following all of the WHO/FAO healthy food recommendations."

[this is a very very brief summary. Please provide a detailed numerical summary to guide citizens, governments and researchers how to remedy the problem]

VERSION 1 - AUTHOR RESPONSE

Reviewer: 1
Dr. Akim Tafadzwa Lukwa, University of Cape Town

Comments to the Author:
Thank you for submitting your manuscript to BMJ Open and allowing me to review your work. The study "Patterns, Socioeconomic Inequalities, and Determinants of Healthy Eating in Kenya: Results from a National Cross-Sectional Survey" addresses a significant and timely public health issue with implications for nutritional policy and intervention in Kenya. The thorough approach to analyzing the determinants of healthy eating within the socio-economic framework of Kenya provides valuable insights that could potentially influence public health strategies and dietary guidelines. Below, I offer some suggestions that could further strengthen the manuscript and enhance its contribution to the literature.

Response: Thank you for your review of our manuscript and your valuable feedback.

Title
• Original Line: 1
• Suggestion: Specify the dietary components in the title to clarify the focus of healthy eating. For example:
o Revised Title: "Patterns, Socioeconomic Inequalities, and Determinants of Fruit and Vegetable Intake in Kenya: A National Cross-Sectional Survey Analysis"
o Rationale: This adjustment clarifies the dietary focus, enhancing relevance and searchability for readers interested in diet-related aspects.

Response: Thank you very much for your suggestion. In this paper, healthy eating was defined using nine WHO/FAO dietary recommendations, with fruit and vegetable intake being just one of these components. Given that we have elaborated on the individual components used to generate the healthy diet index in the methods sections, we believe the use of "healthy diet" in the title is appropriate.

Abstract
• Lines: 31-36
• Modifications:
o Objective: Add specifics to the dietary behaviors studied, such as "This study aims to assess the patterns and socioeconomic determinants of fruit and vegetable intake among Kenyan households."

Response: Thank you for your suggestion. The study's aim is already stated in the last sentence of the Objective. Additionally, as we have stated earlier in the Design and methods

section that the healthy diet index was generated using nine WHO/FAO dietary recommendations, with fruit and vegetable intake being just one of these components. (Page 2, Lines 3-4)

o Methods: Include key statistical tools used, "utilizing principal component analysis and multivariable linear regression."

Response: We have revised the ‘Design and methods’ section to include the analysis methods for generation of the HDI index (principal component analysis) and identifying the determinants of eating healthy (multivariable linear regression). (Page 2, Lines 6-11)

o Results: Briefly highlight significant findings, e.g., "We identified key determinants including socioeconomic status and urban-rural residency differences."
o Rationale: These modifications enhance the abstract's clarity and informativeness, providing a concise snapshot of the study’s scope, methods, and key findings.

Response: We have added the statement “"We identified key determinants including socioeconomic status and urban-rural residency differences." before reporting specific results. We have also specified that the reported variables are ‘significantly’ associated with increases or decreases of the HDI scores. (Page 2, Lines 15-16; Lines 19-20)

Introduction
• Lines: 4-5
• Enhancements:
o Elaborate on prior studies specific to the region to better frame the research within the existing literature.

o Explicitly state the research gap your study addresses, such as limited data on the impact of socioeconomic factors on diet within Kenya.

o Example: "While numerous studies have documented dietary habits in East Africa, few have dissectively analyzed the socioeconomic gradients impacting dietary quality among Kenyan households."

Response: Many thanks for this comment. We have revised the introduction section to highlight the relevant studies within the region. We have also highlighted the evidence gap in Kenya, and the overall contribution of this study. Please see Page 4, paragraph 3.

Methods
• Lines: 5-6
• Details to Add:
o Clarify the sampling methodology, "employing stratified random sampling to ensure diverse representation from different Kenyan counties."

Response: We have edited theSampling strategy section in the Methods and revised the description of the sampling methodology for better clarity. (Page 6, paragraph 2, Lines 4-5)

o Proactively mention methodological constraints that might impact results, like "potential recall bias in dietary self-reporting and limitations in the accuracy of food composition data for local dishes."
o Rationale: Provides greater transparency about the methodology's robustness and its limitations.
Response: Thank you for the comment. These points have been included in the 'Study strengths and limitations' section of the manuscript. (Page 6, Lines 4-5)

Results
• Lines: 13-14
• Enhancements:
o Introduce a summary table of key quantitative findings, such as average HDI scores across demographic groups.

Response: Thanks for the comment. We have added a summary table of key quantitative findings as suggested. Table 1 shows the distribution of HDI scores by gender, residence, and socioeconomic status. We have also added a supplementary table (Supplementary table 5) that presents the average HDI scores for each of the 47 counties in Kenya to supplement the map. (Page 9, Table 1; Supplementary table 5)

o Clarify significance levels in the results discussion, noting specific p-values or confidence intervals where applicable.
o Rationale: Facilitates quick data assimilation and underscores the statistical rigor of the findings.
Response: Thanks for this comment. We have clarified the levels of significance in the results and discussion as advised. (Page 14, paragraph 1)

Discussion
• Lines: 18-19
• Improvements:
o Compare your findings with similar studies globally, discussing similarities or differences and what these might mean in the Kenyan context.

Response: We have revised the discussion section as advised comparing results from high income countries with those in LMICs including the study setting in Kenya. (Page 16, Lines 12-17)

o Address the implications of your findings for public health policy in Kenya, proposing specific interventions or policy adjustments.

Response: We have added two paragraphs at the end of the discussion on policy implications of our study findings. (Page 17, Paragraphs 2-3)

o Rationale: Contextualizes the findings within a broader international scope and links results to practical, actionable public health strategies.

Conclusion
• Lines: 20-21
• Suggested Additions:
o Concisely reiterate main findings and their implications for future research or policy.

Response: Thank you for the comment. We have revised the conclusion section to highlight the main findings from the study and provided recommendations for future research. (Page 18, Lines 15-19)

o Recommend directions for future research, such as longitudinal studies to track dietary pattern changes and health outcomes over time.
o Rationale: Enhances the manuscript's impact by highlighting its implications and encouraging further investigation.

Response: We have added recommendations for future research including the use of longitudinal studies to track dietary changes and outcomes over time. (Page 18, Lines 21-25)

Reviewer: 2
Prof. Roger Thomas, University of Calgary
Comments to the Author:
This is an important study.

Response: Thanks for dedicating time to review the paper and for highlighting the importance of the study.

Introduction

Please provide the search terms and databases for your literature review so that readers can assess the comprehensiveness of your search.

You wrote: "In LMICs, a nutrition transition has been documented, with shifts from traditional diets to more processed and refined foods high in saturated fats, trans fats, sugar, and salt 5-7. Obesity, diabetes, and cardiovascular disease are among the diet-related NCDs that have been linked to this 8,9."

[Your readers will be be very interested in numerical outcomes. Please provide these for comparison to your study].

Response: Thanks for this comment. We have revised the introduction section to highlight the relevant numerical outcomes. (Page 4, Lines 7-10)

You wrote: "Despite the available standards and guidelines, increasing evidence shows poor dietary practices in LMICs 15"

"The rising burden of NCDs in developing countries has led to generation of more evidence on dietary patterns and their drivers 1,17. Studies conducted in high-income countries (HICs) have used a composite healthy diet indicator from WHO healthy diet guidelines to assess dietary patterns in their population 18-20."

[please provide detailed numerical outcome data so readers can understand the dimensions of the problem]

Response: Thanks for this comment. We have revised the introduction section to highlight the relevant numerical outcomes. (Page 4, Paragraph 3)

Methods

You wrote: "A two-stage cluster sampling design was employed to select a representative sample. In the first stage, 2,400 (988 in urban and 1,412 in rural areas) clusters were selected and 16 households were selected in the second stage while a sub-sample of 10 households was conducted for the main KIHBS."

[so no randomisation was used. Please provide numerical data to demonstrate whether your sample reflects accurately the Kenyan population]

Response: Many thanks for this comment. Multi-stage clustered random sampling was used to select a representative sample of the Kenyan population. We have revised the methods section for better clarity of the sampling process under the 'Sampling strategy' section. (Page 6, Paragraph 2)

Results

You wrote: "The average monthly per adult equivalent consumption expenditure was USD 76.5 (SD 75.6) with 57% in the rich and richest category and 25% falling in the poor and poorest categories. Majority of household heads had up to primary level education (65%) and were in union (71%). About half of the household heads were employed while 41% and 11% were self-employed …."

"The findings indicate that a high percentage of Kenyan families were not following all of the WHO/FAO healthy food recommendations."

[this is a very very brief summary. Please provide a detailed numerical summary to guide citizens, governments and researchers how to remedy the problem]

Response: Thank you for your comment. We have added a numerical summary of the number of healthy diet recommendations met by Kenyan households. (Page 10, Lines 28-30)

VERSION 2 - REVIEW

**Reviewer**	**2**
**Name**	**Thomas, Roger**
**Affiliation**	**University of Calgary, Family Medicine**
**Date**	**17-Feb-2025**
**COI**	

Thank you for the requested changes. The manuscript now answers more key questions.
